# Design and construction of a double actuated mechanical speed breaker electricity generator

**DOI:** 10.1016/j.heliyon.2020.e04802

**Published:** 2020-09-14

**Authors:** Jude Ezechi Dara, Christian Munachiso Odazie, Paul Chukwulozie Okolie, Andrew Onyemazuwa Azaka

**Affiliations:** Department of Mechanical Engineering, Nnamdi Azikiwe University, PMB 5025, Awka, Anambra, Nigeria

**Keywords:** Energy, Mechanical engineering, Machine design, Mechanical systems, Non-conventional energy, Speed-breaker, Power generation, Alternative energy sources, Energy recovery

## Abstract

The economy runs on energy and quest for development means that more energy is needed. Statistics show that the majority of energy comes from burning fossil fuels, and they pollute our environment. This work is aimed at producing a mechanical speed breaker electricity generator that operates on two actuating strokes. It converts the linear motion of the rack gear that is depressed by the vehicular movement to rotary motion of the pinion gear keyed on the shaft and alternator. The machine gets actuated by compression and release of the springs, thus increasing the output of the machine. It consists of a stable frame, dome-shaped top, rack and pinion mechanism, chain and sprocket mechanism, freewheels, springs, flywheel, shafts, an alternator and a deep cycle battery. The design was carried out using engineering principles with due consideration to cost, serviceability, ease of operation, durability and performance. It is designed to be actuated by vehicles weighing 1,600kg (400kg per wheel) and a prototype rated 100kg was built. Tests were performed to determine the performance of the machine and the results showed that the speed breaker could produce a rotary speed of 1500 rpm at the alternator, thereby generating 204W of electrical power per cycle. The machine conversion efficiency was about 88.7%.

## 1 Introduction

The fundamental principle of electricity generation was discovered in the 1820s and early 1830s by the British scientist Michael Faraday. His basic method is still used today; electricity is generated by the movement of a loop of wire or disc of copper between the poles of a magnet. The continuous supply of the drive needed to move this loop of wire or disc of copper lies our energy problems. This drive is often generated by heat engines fuelled by chemical combustion or nuclear fission. Various estimates have been made on when these fuels will be exhausted. New sources of fossil fuels keep being discovered, although the rate of discovery is slow compared with exploration and the difficulty of green extraction is increasing (Institution of Mechanical Engineers, 2019). Another concern is the emissions that result from the burning of fossil fuel as they pollute our environment. The estimated carbon dioxide emission from the world's electrical power industry is 10 billion tonnes yearly (Centre for Global Development, 2007).

Because of aforementioned concerns, there is need to explore other alternative sources of energy. Significant work has been done on renewable energy systems, but they are still unpredictable (wind sources), and unavailable all day round (solar energy) (State Impact Pennsylvania, 2012). An exciting alternative is to use mechanical speed-breakers electricity generators to convert kinetic energy from vehicular movement to electrical energy. The speed-breaker assembly absorbs energy from vehicular movement, while the linear-rotary motion transformation mechanism converts it to rotary motion for the alternator. The machine is best installed on roads with high vehicular movements that require traffic calming.

Attempts have been made to harness energy from moving vehicles with mechanical speed-breakers using different linear-rotary motion transformation mechanisms. They include: Rack and Pinion mechanism, Roller Mechanism and Crankshaft Mechanism (Aswathaman and Priyadharshini, 2010; Mishra et al., 2013; Rao et al., 2014; Azam et al., 2016; Teja et al., 2016).

Santosh et al. (2014) designed their machine using the roller mechanism. In their design, metal tubes known as rollers were arranged in a parallel fashion and fastened to the frame with rolling contact bearings. The rollers used were made with EN8. EN8 is an unalloyed medium carbon steel with good tensile strength. The rollers are connected to a generator to convert the kinetic energy into mechanical energy. Teja et al. (2016) observed that the roller mechanism was challenging to maintain and was plagued with collision problems. Teja et al. (2016) also opined that the crank-shaft mechanism had balancing issues, and it was prone to mechanical vibration.

Previously, some authors have explored the rack and pinion mechanism. A rack and pinion is a type of linear actuator that comprises a pair of gears which convert rotational motion into linear motion. A circular gear called “the pinion” engages teeth on a linear gear bar called "the rack"; linear motion applied to the rack causes the pinion to move relative to the rack, thereby translating the linear motion of the pinion into rotational motion. For every pair of the conjugate involute profile, there is a basic rack and this basic rack is the profile of the conjugate gear of infinite pitch radius (i.e., a toothed straight edge) (American Gear Manufacturers Association, 2005).

Aswathaman and Priyadharshini (2010), Mishra et al. (2013) and Rao et al. (2014) designed rack and pinion mechanical speed breakers electricity generators and recorded outputs of 245watts for 250kg load, 441watts for 300kg load, and 147watts for 150kg load respectively in an hour. Their designs did not harness the energy stored in the springs during compression. The design and construction of a double actuated mechanical speed-breaker are pertinent as it harnesses the energy stored in the spring and increases the machine output significantly.

## 2 Materials and method

### 2.1 Machine description

**Figure 1:**
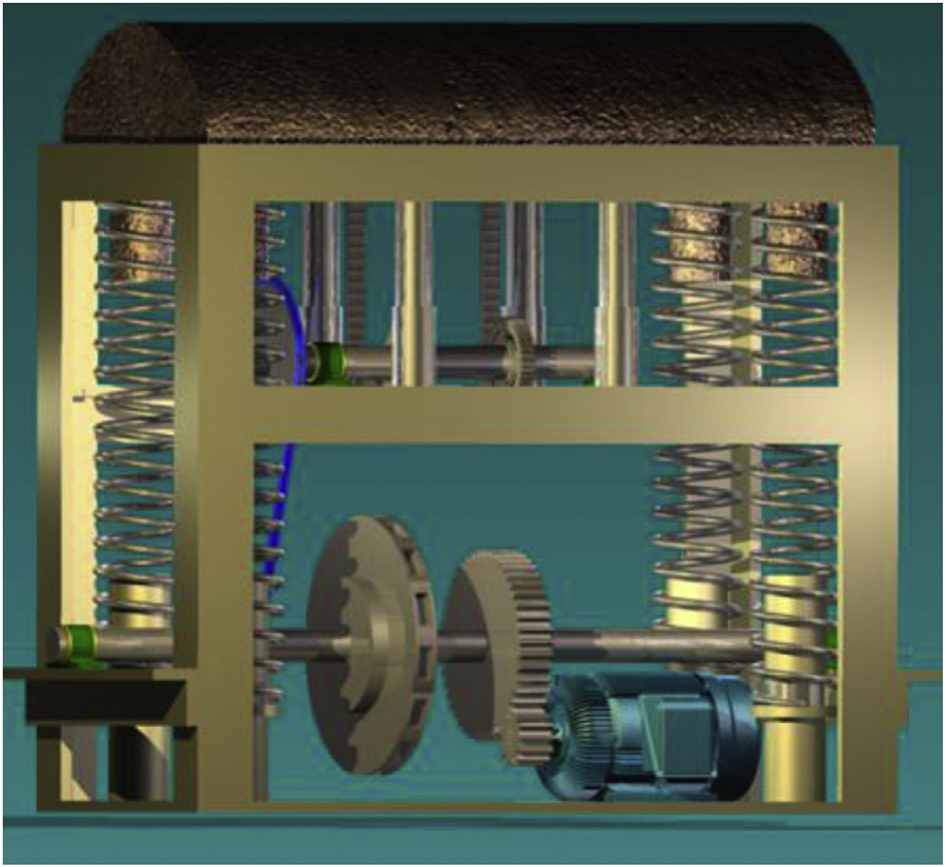
Model of the speed breaker assembly.

The speed breaker is a stable frame structure that consists of various components such as rack and pinion gears, flywheel, chain and sprocket, properly mounted on shafts and some other components such as journal bearings, helical springs and a dome-shaped hump (see Figure 1).

### 2.2 Material selection

Good material selection was undertaken to reduce noise and wear, ease of maintenance, improve service life and reduce corrosion.

Machine frame, shaft and dome-shaped top – (Mild Steel).

• Carbon content ranges from 0.1 -0.3% and iron content of 99.7–99.9%.
• Offers an excellent weldability
• It has a good balance of toughness, strength and ductility.
• Improved machining characteristics.
• Mild steel is highly malleable and can be used for various construction purposes.

Springs – (Oil Tempered Carbon Steel).

• Quenched and tempered to improve toughness and ductility.

Rack and pinion gear mechanism – (Cast iron).

• Good wear resistance.
• Resistance to corrosion.

### 2.3 Machine design

**Figure 2:**
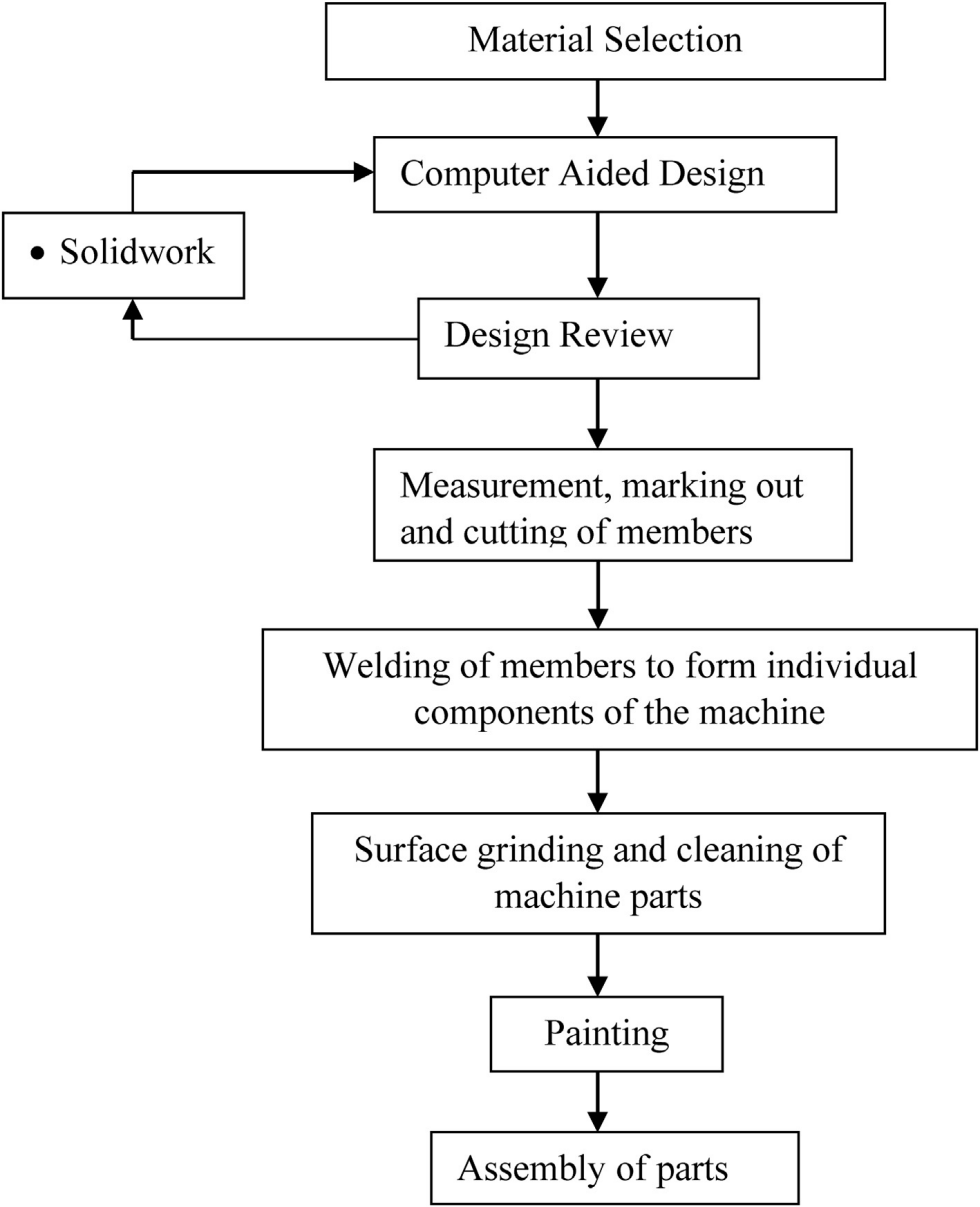
Design process flow chart.

Institute of Transport Engineers (1997) published recommended practice for the design of normal speed-breakers. Parkhill et al. (2007) opined that most agencies recommend that the height of speed breakers be between 76.2 and 152.4mm. This machine will be designed with a height of 101.4mm for the safety of vehicles and passengers. Figure 2 presented the flow chart for the design process.

#### 2.3.1 Design of spring

According to Khurmi and Gupta (2005), the following points should be considered when designing springs.

• The spring should not close up before the maximum service load is reached.
• Failure is due to the twisting moment (T) set up in the wire. Thus, torsional stress is induced in the wire.

The pitch of the coil should be such that if the spring is accidentally or carelessly compressed, the stress does not increase beyond the yield point stress in torsion. The design parameters for the spring are presented in Table 1. Relationships for the design of spring, according to Khurmi and Gupta (2005):

(1) $$\text{Solid}\;\text{length,}\;\text{LS}=\;n^{'}.{\text{d}}_{\text{sp}}$$

(2) $$\text{Free}\;\text{Length,}\;\text{LF}=\;n^{'}.d_{sp}+{\text{δ}}_{\text{max}}+0.15\;{\text{δ}}_{\text{max}}$$

(3) $$\text{Spring}\;\text{index,}\;\text{C}=\frac{D}{d_{sp}}$$

(4) $$\text{Spring}\;\text{Rate/Spring}\;\text{Constant,}\;\text{k}=\frac{W}{\delta }$$

(5) $$\text{Pitch,}\;\text{p}=\frac{Free\;Length}{n^{'}-1}=\frac{L_{F}-L_{S}}{n^{'}}+\text{dsp}$$

(6) $$\text{Load/Cross}-\text{sectional}\;\text{area}\;\text{of}\;\text{the}\;\text{wire,}\tau_{2}=\frac{W}{\frac{\pi }{4}\times d^{2}\;}=\frac{4W}{\pi d^{2}\;}$$

**Table 1:** Designed Spring Parameters Using spreadsheet and relationships by Khurmi and Gupta (2005).

Parameters	Prototype (100 kg)	Product (400 kg)	Symbols	Units
Spring Coil Diameter	70.00	180.00	D	mm
Spring Wire Diameter	8.00	15.00	d	mm
Total Number of Coils	18.00	18.00	n'	
Solid Length of Spring	144.00	270.00	Ls	mm
Free Length of Spring	450.00	450.00	Lf	mm
Spring Index	8.75	12.00	C	
Spring Deflection	0.10	0.10	δ	m
Weight/Load on Spring	1000.00	4000.00	W	N
Spring Rate/Spring Constant	10000.00	40000.00	k	N/m
Pitch	25.00	25.00	p	mm
Shear Stress Factor	1.06	1.04	Ks	
Wahl's Stress Factor	1.17	1.12	K	
Maximum Shear Stress	368.00	565.81	τ	MPa
Energy stored in the spring	50.00	200.00	Es	J

Resultant shear stress induced in the wire

(7) $$\tau =8\text{W}\times \text{D/}\pi {\text{d}}^{3}\pm 4\text{W/}\pi {\text{d}}^{2}$$

Maximum shear stress induced in the wire

(8) τ_max_ = 8W.D/πd^3^ (1 + (1/2C))

where, C = D/d

(9) K_S_ = 1 + (1/2C)

(10) τ_max_ = K x 8W.D/πd^3^

(11) K = ((4C–1)/ (4C–4)) + (0.615/C)

#### 2.3.2 Design of shafts

When a shaft is subjected to twisting and bending moment, the resultant stresses should not exceed the allowable stress. The design parameters for the shafts are presented in Tables 2a and 2b. Relationships for design of shaft according to Khurmi and Gupta (2005):

(12) P.E = Mgδ

(13) E_sp_ = 0.5kδ^2^

(14) E_sh_ = PE-E_sp_-E_f_

(15) T = (P×60)/2πN

(16) F_t_ = 2T/D

(17) T_e_ = √(M^2^+T^2^)

**Table 2a:** Designed Shaft-1 Parameters Using spreadsheet and relationships by Khurmi and Gupta (2005).

Parameters	Prototype (100 kg)	Product (400 kg)	Symbols	Units
Mass	100	400	M	kg
Deflection	0.1	0.1	δ	m
Acceleration due to gravity	10	10	g	m/s^2^
Potential energy available	100.00	400.00	P.E	J
Energy Stored in the spring	50.00	200.00	Es	J
Energy transmitted to the shaft	50.00	200.00	Esh	J
Duration of compression	0.15	0.15	t	s
Power transmitted by the shaft	333.33	1333.33	Psh	W
Speed of the shaft	200.00	200	N	rpm
Desired length of shaft	0.31	0.30	L	m
Torque transmitted by shaft	15.91	63.65	T	N.m
Load on the gear tooth	445.65	1782.60	W	N
Max bending moment from the chain	18.45	60	m1	N.m
Max bending moment from gear 1	23.70	80	m2	N.m
Max bending moment from gear 2	100.00	120	m3	N.m
Maximum bending moment	100.00	120.00	mt	N.m
Factor of safety	1.25	1.25		
Allowable shear stress	45.00	45.00	τ	MPa
Equivalent twisting moment	101.26	135.84	Te	N.m
Diameter of the shaft	24.29	26.78	d	mm

**Table 2b:** Designed Shaft-2 Parameters Using spreadsheet and relationships by Khurmi and Gupta (2005).

Parameters	Prototype (100 kg)	Product (400 kg)	Symbols	Units
Power transmitted by the shaft	333.33	933.33	Psh	W
Speed of the shaft	500.00	500	N_2_	rpm
Desired length of shaft	0.31	0.31	L_2_	m
Torque transmitted by shaft	6.37	17.82	T	N.m
Diameter of the gear 3	0.17	0.17		m
Weight of fly wheel	5.00	5.00		kg
Load on the gear tooth	74.89	209.68	W	N
Max bending moment from the chain	21.70	75.95	m1	N.m
Max bending moment from gear	34.80	121.8	m2	N.m
Max bending moment from flywheel	52.08	182.28	m3	N.m
Maximum bending moment	52.08	182.28	mT	N.m
Factor of safety	1.50	1.50		
Allowable shear stress	45.00	45.00	τ	MPa
Diameter of the shaft	20.68	31.39	d2	mm

#### 2.3.3 Design of chain drive

Speed of the smaller sprocket = 240 rpm.

Desired velocity ratio = 4.

From Table 3;

**Table 3:** Number of teeth on the smaller sprocket.

Type of chain	Number of teeth at velocity ratio					
	1	2	3	4	5	6
Roller	31	27	25	23	21	17
Silent	40	35	31	27	23	19

Number of teeth on the small sprocket, T_1_ = 23.

Number of teeth on the larger sprocket, T_2_ = 23X4 = 92

(18) SF = LF (Luf) Rf

SF = 1.25X1.5X1 = 1.875

Design power = Rated power x Service factor = 1.875X150 = 281W

From standard table, for speed below 300rpm, the type of simple roller chain recommended is 06B and the parameters of the chain that corresponds to 06B are.

Pitch = 9.5mm; Roller Diameter = 6.35 mm; Width thickness between inner plates = 5.72mm; Transverse pitch = 10.24mm; Breaking load = 8.9KN.

Pitch circle diameter of smaller sprocket, d_1_ = $\;p\;cosec\left(\frac{180}{T1}\right)$ cosec 180/T1 = $9.5\;cosec\;\left(\frac{180}{23}\right)$ cosec 180/23 = 0.07m.

Pitch circle diameter of larger sprocket, d_2_ = $\;p\;cosec\left(\frac{180}{T2}\right)$ cosec 180/T2 = $9.5\;cosec\left(\frac{180}{92}\right)$cosec 180/92 = 0.278m.

Pitch line velocity on the smaller sprocket, v_1_ = $\frac{\pi d1N1}{60}$ = $\frac{\pi \times 0.07\times 240}{60}$ = 0.88 m/s.

∴ Load on the chain, W = $\frac{Rated\;power}{Pitch\;line\;velocity}$ = $\frac{75}{0.88}$ = 85.2N.

Factor of safety = $\frac{W_{b}}{W}=\frac{8.9\times 1000}{85.2}$ = 104.

The factor of safety is large; this means that the chain is very safe.

The minimum centre distance, x = 30p = (9.5)30 = 285mm.

To correct initial sag, x = 285–3 = 282mm

(19) $$\text{K}=\frac{T1+T2}{2}+\frac{2x}{p}+{\left[\frac{T2-T1}{2\pi }\right]}^{2}\frac{p}{x}$$

(20) L = k∗p

#### 2.3.4 Design of gear

In the design of gear, the common normal at the point of contact between a pair of teeth must always pass through the pitch point (Khurmi and Gupta, 2005).

According to Khurmi and Gupta (2005), to prevent interference, the addendum circles of the two mating gears must cut the common tangent to the base circles between the points of tangency. Thus, the minimum number of teeth on the pinion (T_P_) in order to avoid interference may be obtained from the relation:

(21) $$T_{p}=\frac{2aw}{G\left[\sqrt{1+\frac{1}{G}}\left(\frac{1}{G}+2\right)\times sin^{2}\emptyset -1\right]}$$

(22) $$\text{Torque,}\;\text{T}=\frac{60*P}{2*\pi *N}$$

Thus, choosing a standard module (m) of 2 for the gear, considering the power to be transmitted,

(23) $$\text{m}=\frac{D}{T}$$

(24) $$\text{TP}=\frac{D_{p}}{m}$$

(25) $$\text{Pd}=\frac{1}{m}=\frac{T_{P}}{D_{P}}$$

(26) aw = 1(m)

(27) dw = 1.25(m)

D’ = D_P_+(2 X aw) (28)

(29) D = D_P_-(2 X dw)

(30) $${\text{C}}_{\text{t}}={\text{D}}_{\text{P}}\text{Xsin}\pi \text{X}\left(\frac{90}{T_{P}}\right)\;\times \left(\frac{1}{180}\right)$$

(31) $$\text{Cit}=\left(\frac{\pi }{2}\right)\times \left(\frac{1}{P_{d}}\right)$$

(32) $$\text{Pc}=\frac{\pi D_{P}}{T_{P}}$$

(33) C_l_ = (dw – aw)

(34) (φ) = 20^o^

(35) $${\text{D}}_{\text{b}}={\text{D}}_{\text{p}}\text{X}\left\{\text{cos}\pi \;\text{X}\left(\frac{\varphi }{180}\right)\right\}$$

(36) D_w_ = 2(m)

(37) MTD = 2.25(m)

(38) FR = 0.4(m)

### 2.4 Principle of operation

**Figure 3:**
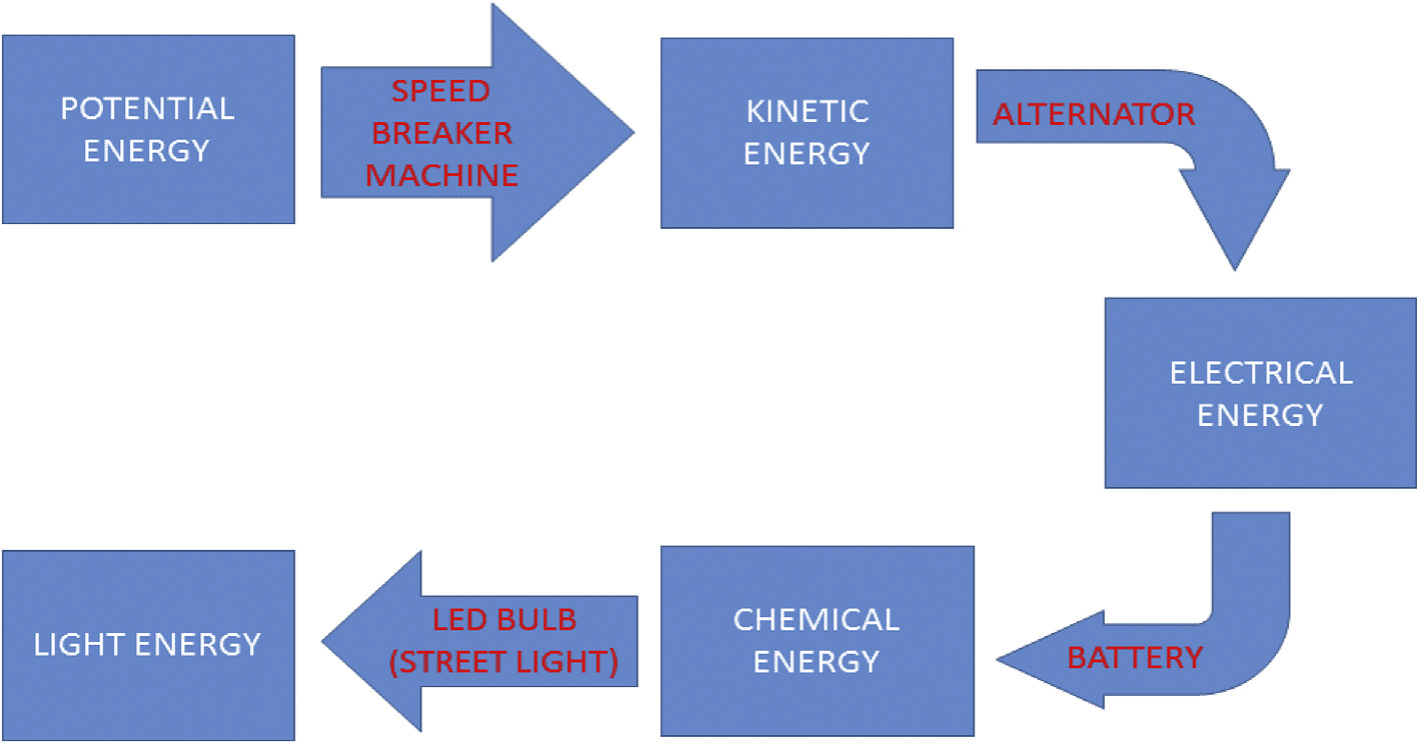
Speed breaker energy conversion diagram.

**Figure 4:**
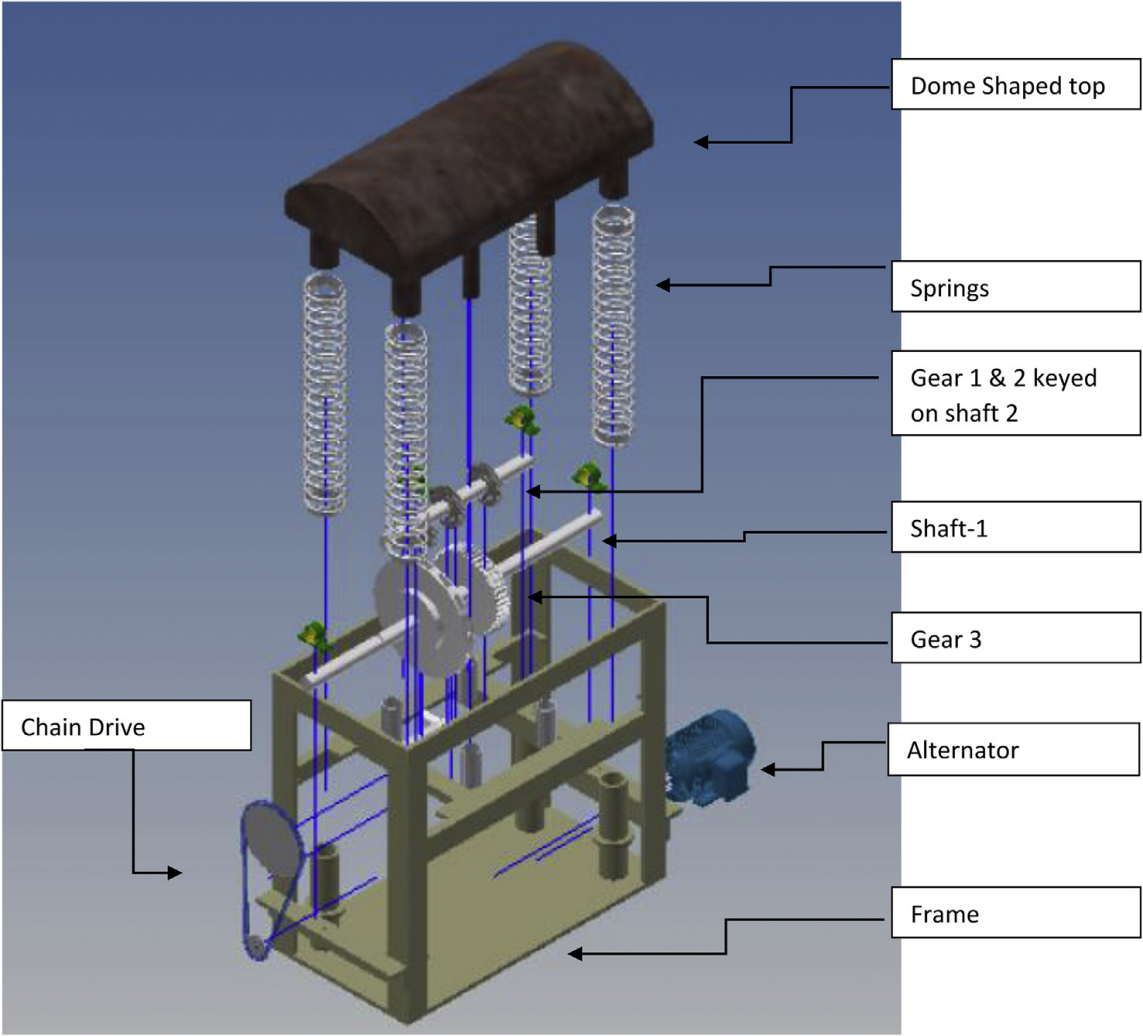
Exploded view of the speed breaker assembly.

**Figure 5:**
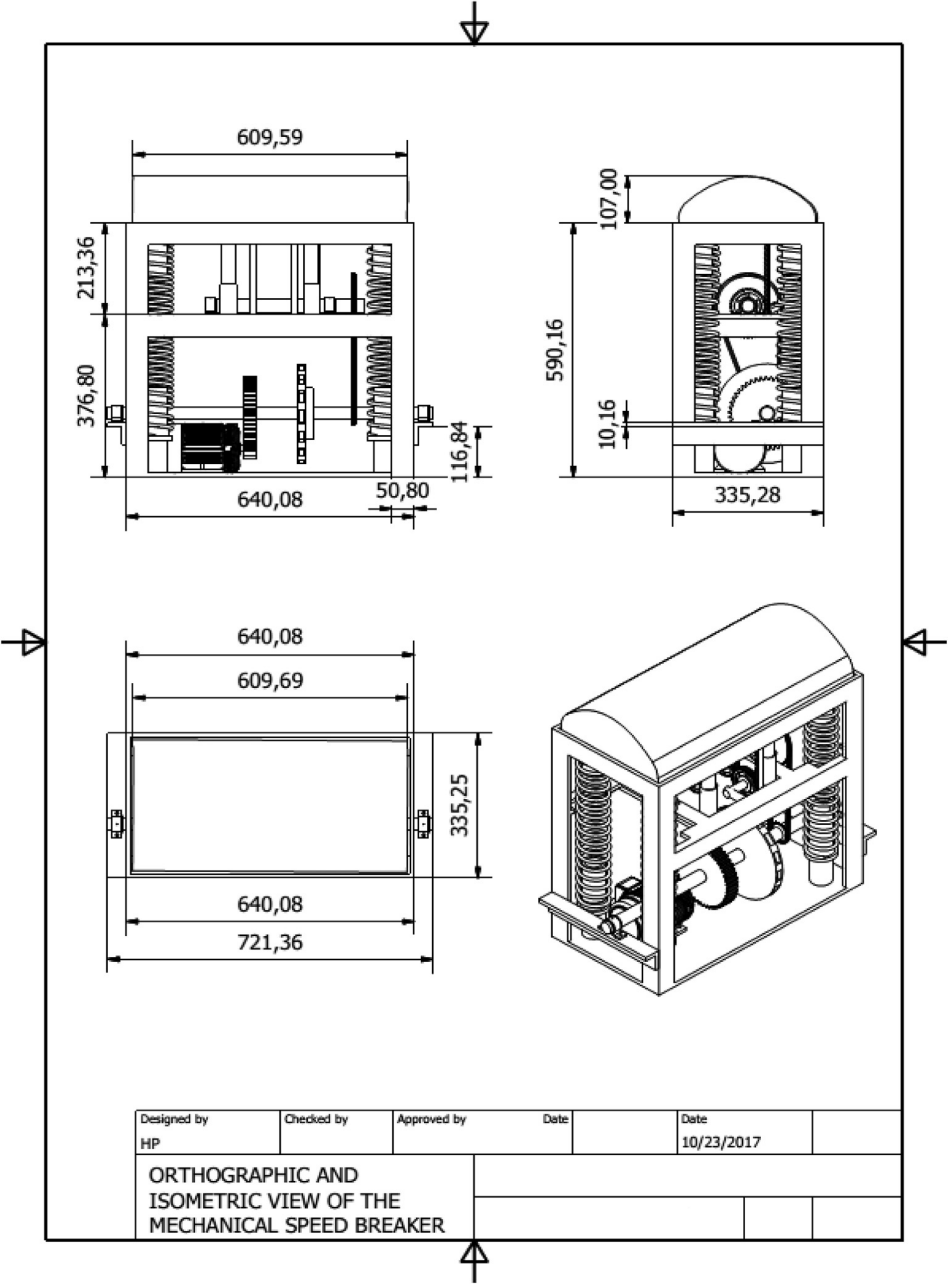
Orthographic and isometric view of the Speed Breaker Assembly (all dimensions in mm).

When a moving vehicle climbs the speed breaker, the springs of the speed breaker get compressed as the dome is depressed. Some energy from the vehicle is converted to rotary motion by the rack and pinion mechanism and sent to the shaft while the rest is stored in the springs. This rotary motion undergoes speed multiplication in the chain drive and the spur gears before it is fed to the alternator. As the vehicle exits the speed breaker, the springs begin to return to their original shape thereby releasing their stored energy to the rack and pinion gears of shaft 2 for conversion to rotary motion. This rotary motion also undergoes speed multiplication in the chain drive and spur gears before it is sent to the alternator. In this machine, electrical energy is produced during the compression and return strokes, hence providing more energy. The operation principle is illustrated in Figure 3, while Figures 4 and 5 presented various views of the machine.

## 3 Results and discussions

### 3.1 Design results for the mechanical speed breaker electricity generator

The results of the design of the mechanical components of the speed breaker were presented in Tables 1, 2a, 2b, 3, 4, 5, 6, 7, and 8. Table 1 shows the design parameters for the spring, Tables 2aand 2b present the design parameters for the two shafts that carry the rotating members of the generator. Tables 3, 4, 5, and 6 show the design parameters for the flexible drive elements of the generator, while Tables 7 and 8 show the design parameters for the gear drives.

**Table 4:** Power Rating (in kW) of Simple Roller Chain.

Speed of smaller sprocket (rpm)	Power (kW)				
	06B	08B	10B	12B	16B
100	0.25	0.64	1.18	2.01	4.83
200	0.47	1.18	2.19	3.75	8.94
300	0.61	1.70	3.15	5.43	13.06
500	1.09	2.72	5.01	8.53	20.57
700	1.48	3.66	6.71	11.63	27.73
1000	2.03	5.09	8.97	15.65	34.89
1400	2.73	6.81	11.67	18.15	38.47
1800	3.44	8.10	13.03	19.85	-
2000	3.80	8.67	13.49	20.57	-

**Table 5:** Characteristics of roller chains according to is: 2403-1991.

ISO Chain number	Pitch (p) mm	Roller diameter (d_1_) mm maximum	Width between inner plates (b_1_) mm maximum	Transverse pitch (p_1_) mm	Breaking load (kN) minimum		
					Simple	Duplex	Triplex
05B	8.00	5.00	3.00	5.64	4.4	7.8	11.1
06B	9.525	6.35	5.72	10.24	8.9	16.9	24.9
08B	12.70	8.51	7.75	13.92	17.8	31.1	44.5
10B	15.875	10.16	9.65	16.59	22.2	44.5	66.7
12B	19.05	12.07	11.68	19.46	28.9	57.8	86.7
16B	25.4	15.88	17.02	31.88	42.3	84.5	126.8
20B	31.75	19.05	19.56	36.45	64.5	129	193.5
24B	38.10	25.40	25.40	48.36	97.9	195.7	293.6
28B	44.45	27.94	30.99	59.56	129	258	387
32B	50.80	29.21	30.99	68.55	169	338	507.10
40B	63.50	39.37	38.10	72.29	262.4	524.9	787.3
48B	76.20	48.26	45.72	92.21	400.3	400.3	1201

**Table 6:** Designed chain Parameters Using spreadsheet and relationships by Khurmi and Gupta (2005).

Parameters	Prototype (100 kg)	Product (400 kg)	Symbols	Units
Power to be transmitted	333.33	933.33	P	W
Speed of the larger sprocket	200.00	200	N_1_	rpm
Speed of the smaller sprocket	600.00	600	N_2_	rpm
Velocity ratio	3.00	3.00		
Number of teeth on the larger sprocket	42.00	42.00	T_1_	
Number of teeth on the smaller sprocket	18.00	14.00	T_2_	
Load factor	1.25	1.25		
Lubrication factor	1.50	1.50		
Rating factor	1.00	1.00		
Service factor	1.88	1.88		
Pitch	13.00	13.00	*p*	mm
Breaking load	8.90	8.90	W_b_	kN
Pitch circle diameter of smaller sprocket	0.07	0.06	d_1_	m
Pitch circle diameter of larger sprocket	0.17	0.17	d_2_	m
Pitch line velocity on the smaller sprocket	0.78	0.61	v_1_	m/s
Load on the chain	425.18	1525.57	W	N
Factor of safety	20.93	5.83	F.S	
Minimum centre distance	390.00	390.00	x_min_	mm
Desired centre distance	250.00	250.00	x	mm
Number of links	69	67	K	
Length of chain	899.86	877.42	L	mm

**Table 7:** Designed Parameters of Gear 1, Gear 2 and Gear 3 (prototype 100 kg) according to Khurmi and Gupta (2005) using a spreadsheet.

Parameters			Values	Units
Load on Gear, M			100	kg
Acceleration Due to Gravity, g			10	N/m^2^
Height, h			0.15	m
Potential Energy, PE			150	J
Power, P			150	W
Speed of Shaft, N			60	rpm
Torque Transmitted to the Pinion, T			23.87015	N-m
Standard Module of Gear, m			2	

Parameters	Gear 1	Gear 2	Gear 3	
Pitch Diameter, Dp	76.2	34	170	mm
Number of Teeth, Tp	38.1	17	85	
Diameteral Pitch, Pd	0.5	0.5	0.5	
Addendum, aw	2	2	2	mm
Dedendum, dw	2.5	2.5	2.5	mm
Outside Diameter, D′	80.2	38	174	mm
Root Diameter, d	71.2	29	165	mm
Chordal Thickness	3.140703	3.137124	3.141414	mm
Circular Thickness	3.142	3.142	3.142	mm
Circular Pitch, Pc	6.284	6.284	6.284	mm
Clearance	0.5	0.5	0.5	mm
Pressure Angle, ϕ	20	20	20	deg.
Base Diameter	76.2	34	170	mm
Working Depth	4	4	4	mm
Minimum Total Depth	4.5	4.5	4.5	mm
Fillet Radius	0.8	0.8	0.8	mm
Face Width, w	25.4	25.4	25.4	mm

**Table 8:** Designed Parameters of Gear 1, Gear 2 and Gear 3 (product 400 kg) according to Khurmi and Gupta (2005) using a spreadsheet.

Parameters			Values	Units
Load on Gear, M			400	kg
Acceleration Due to Gravity, g			10	N/m^2^
Height, h			0.15	m
Potential Energy, PE			600	J
Power, P			600	W
Speed of Shaft, N			60	rpm
Torque Transmitted to the Pinion, T			95.48059	N-m
Standard Module of Gear, m			2	

Parameters	Gear 1	Gear 2	Gear 3	
Pitch Diameter, Dp	76.2	34	170	mm
Number of Teeth, Tp	38.1	17	85	
Diameteral Pitch, Pd	0.5	0.5	0.5	
Addendum, aw	2	2	2	mm
Dedendum, dw	2.5	2.5	2.5	mm
Outside Diameter, D′	80.2	38	174	mm
Root Diameter, d	71.2	29	165	mm
Chord Thickness	3.140703	3.137124	3.141414	mm
Circular Thickness	3.142	3.142	3.142	mm
Circular Pitch, Pc	6.284	6.284	6.284	mm
Clearance	0.5	0.5	0.5	mm
Pressure Angle, ϕ	20	20	20	deg.
Base Diameter	76.2	34	170	mm
Working Depth	4	4	4	mm
Minimum Total Depth	4.5	4.5	4.5	mm
Fillet Radius	0.8	0.8	0.8	mm
Face Width, w	25.4	25.4	25.4	mm

It was observed that vibration was within safe limits from the vehicular speed of 1 km/h to 5 km/h and the springs performed in line with the design. As the speed increased beyond the designed speed of 5 km/h, vibrations began to significantly affect the performance of the machine. Therefore, the breaking speed of the vehicle must be considered in the design of the mechanical speed breaker for electricity generation.

### 3.2 Performance evaluation of the mechanical speed breaker electricity generator

After designing and building the mechanical speed breaker for electricity generation, the necessary tests were performed on the machine to determine its performance. The result of the application of a single load at different speeds is presented in Table 9, and the result of the application of different test loads at the same speed is presented in Table 10.

**Table 9:** Results of applying a moving load of 100 kg at various speeds.

Speed of the load (km/hr.)	Main shaft peak speed (rpm)	Duration of shaft rotation (hr.)
1	130	1.0
2	135	1.2
3	150	1.2
4	152	1.4
5	180	1.5
6	182	1.5
7	180	1.4
8	170	1.3
9	171	1.4
10	170	1.3

**Table 10:** Results of applying varying moving loads at a constant speed of 5 km/h.

Test load (kg)	Spring deflection(m)	Main shaft peak speed
60	0.06	112
80	0.08	159
100	0.10	180
120	0.12	188
140	0.14	180
160	0.16	183

These runs are necessary to have complete knowledge of what happens in the system. It is also essential to determine the power generated by the machine. The generator was tested with a moving load of 100 kg at different speeds. The following were taken into considerations during the test.

• The generator was appropriately secured on a stable surface
• The movement of the load was perpendicular to the axis of the machine
• The load was applied such that all the springs were equally loaded.
• All measuring instruments were appropriately calibrated before the test.

**Figure 6:**
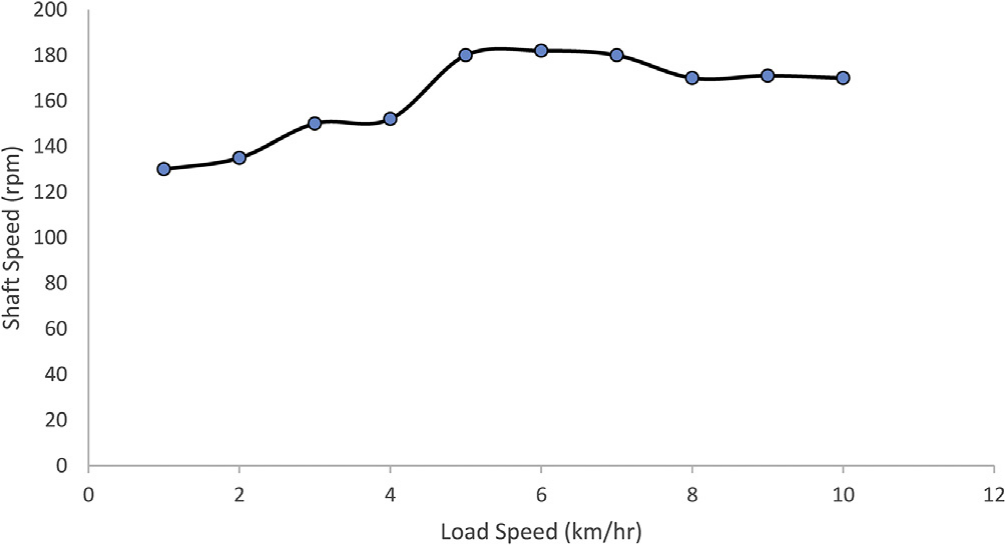
A graph of shaft speed against load speed.

Figure 6 depicts the rotational speed of the generator with varying load speed. It was observed that the rotation speed of the generator increased with load speed up to 5 km/h. The machine performed as designed at load speed below 6 km/h Figure 6 also shows that the peak shaft speed of 182rpm occurred between 5 and 6 km/h, indicating that the machine is more efficient when the speed of moving vehicle is within 5–6 km/h. When considering the testing load of 100kg, the applied force, F on the generator, is 981N. The angular velocity, *ω,* is given in Eq. (41) and linear velocity, *v* is given in Eq. (40). For the peak shaft speed of 182rpm, the angular velocity is 19.06sec^−1^. The diameter of the output shaft is 24.29mm; therefore, the linear velocity is 0.2315 m/s Eq. (39) gave the power output for 100kg of load with a speed of 5 km/h as 227.1W. Moreover, when power was sent to the alternator, a potential difference of 12V was recorded across the terminals of the battery, with a current of 17A flowing through it. It implies that 204 W of electrical power was produced using Eq. (42). It can be seen that electrical power is less than the mechanical power as a result of conversion losses. The conversion efficiency of the machine is about 88.7%. This high conversion efficiency was achievable as a result of double actuation employed in this design and power was sent to the alternator on both operating strokes. This indicates that as a result of the double actuating, the machine converts 88.7% of mechanical energy into electrical energy. Electricity is a secondary energy source that must be harnessed from the primary source. The conversion from primary sources to electrical energy is usually associated with losses. In most cases, the conversion process to electrical energy goes through intermediary stages. The present machine converts mechanical energy directly to electrical energy with minimal losses.

(39) P=Fv

(40) v=rω

(41) $$\omega =\frac{2\pi N}{60}$$

(42) P=VI

a constant vehicular speed of 5 km/h and varying test loads of 60, 80, 100, 120, 140 and 160kg. The following considerations were taking in the test runs:

• The machine was secured firmly on a stable surface
• All measuring instruments were tested and appropriately calibrated before the test.
• The movement of the load was perpendicular to the axis of the machine
• The load was applied such that all the springs were equally loaded.
• When testing the exceeding designed load, the load was concentrated over a large area.

**Figure 7:**
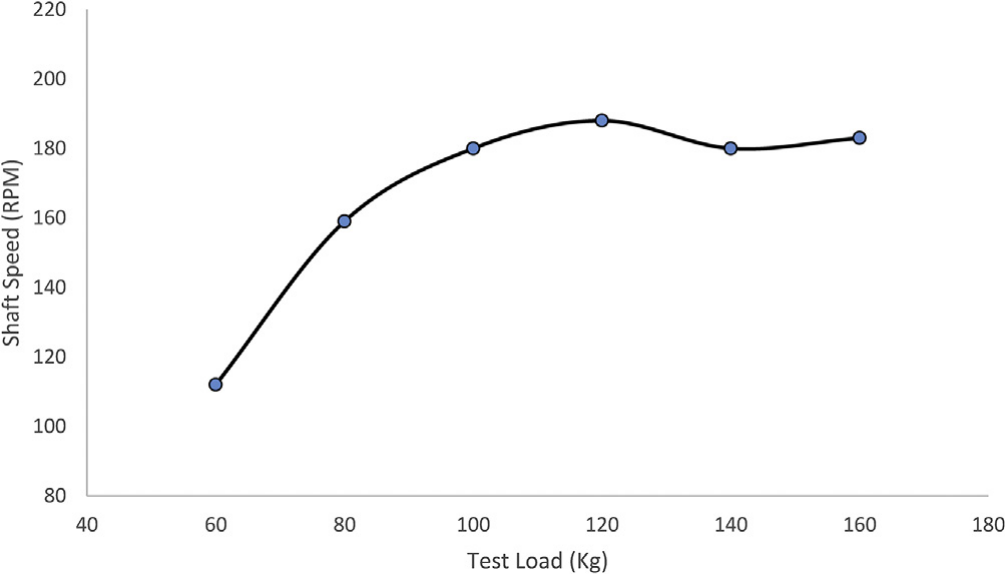
A graph of shaft speed against test load.

Figure 7 depicts the variation of the speed of the alternator's shaft with the load. It showed that shaft speed recorded a maximum value of 188rpm when the test load ranged between 100 and 120 kg. When the test load exceeded this range, the machine experienced vibration with the springs deflecting beyond design limits. There was a linear relationship between the test load and shaft speed below the load of 100kg. As the load increases, the rotational speed of the shaft also increases. Heavier vehicles will prompt more significant deflection of the spring and consequently, high rotational speed. The drop in performance during overload was as a result of excessive vibrations. The deflections of the springs were between 7 and 8cm when the loads were below 100kg. A deflection of 10cm was recorded between 100 and 120kg, and the springs performed as according to the design. The machine performed better at the designed load compared to other loads. Institute of Transport Engineers (1997) and Parkhill et al. (2007) recommended that the height of speed-breakers be between 7.62cm and 15.2cm to ensure the comfort of motorists. Therefore, the deflection of 10cm recorded with the designed test load is within the acceptable limit.

**Figure 8:**
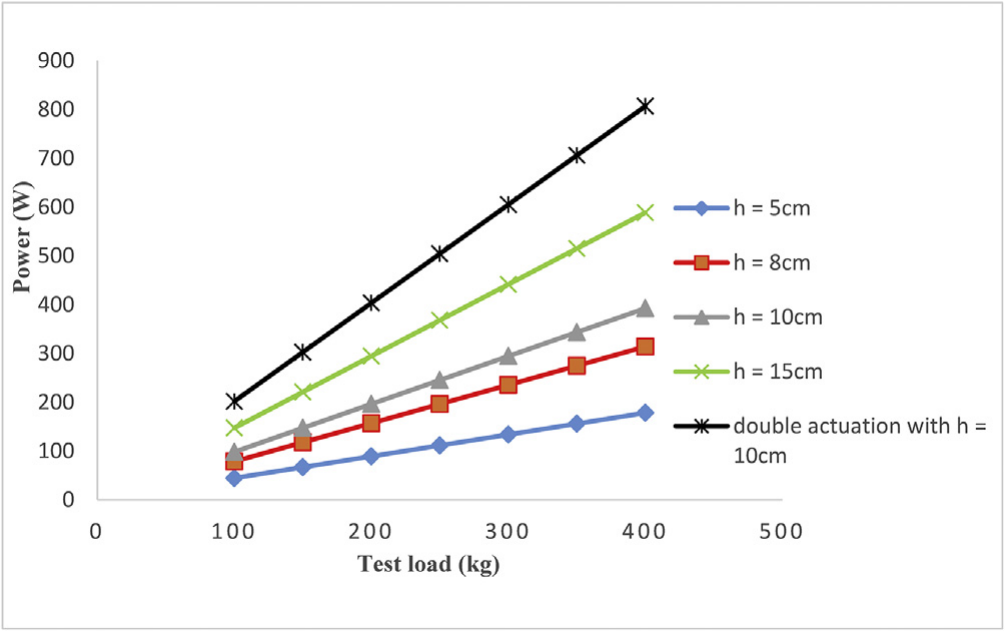
A graph of power generated against test load.

Figure 8 shows the variation of the electrical power output of the speed breaker with loads for different design schemes. Different loads were applied to the mechanical speed breaker and generated power measured. It was observed that a load of 400kg can be generated up to 806.66W with this new speed breaker for power generation. The results obtained were compared with the results of previous works. There is a linear relationship between electrical power and test load, as depicted in Figure 8. The power increases as the load increases, showing that the size of the vehicle will determine the amount of electricity to be produced. Another factor identified to influence the amount of electricity to be produced is the height of the speed breaker, which is the distance the vehicle will depress the springs upon rolling over them. A higher speed breaker will produce a more significant amount of electricity. Furthermore, 10cm is usually taken as the height of a standard speed breaker for the comfort and convenience of motorists (Institute of Transport Engineers, 1997; Parkhill et al., 2007). Many researchers have developed speed breaker electricity generators using a standard height of 10cm, shown in Figure 8 as “h = 10cm” (Aswathaman and Priyadharshini, 2010; Rao et al., 2014). Santosh et al. (2014) developed theirs using a height of 5cm represented in Figure 8 as “h = 5cm”. Fawade (2015) used a height of 8cm represented in Figure 8 as “h = 8cm”; while Mishra et al. (2013) worked with a height of 15cm represented in Figure 8 as “h = 15cm”. The machines developed by Aswathaman and Priyadharshini (2010) and Rao et al., 2014 can produce power of 392.4W when a test load of 400kg is applied. The machine developed by Santosh et al. (2014) can generate 178.2W of power from the same 400kg test load whereas that developed by Fawade (2015) has the capability to generate electrical power 313.92W when a test load of 400kg is applied. Mishra et al. (2013) produced a machine that can generate electrical power of 588.6W with an applied test load of 400kg. The present design developed the machine using 10cm as the height of the speed breaker and is depicted in Figure 8 as “double actuation with h = 10cm”. The present design produced power of about 105% higher than identical mechanisms with a maximum height of 10cm operating only on the compression stroke. It also produced power of about 37% higher than a speed breaker with a maximum height of 15cm. Utilizing the strain energy stored in the spring during the compression stroke to turn the generator during retraction stroke is an effective means of improving the electricity generation using a mechanical speed breaker. The present design utilized both compression and retraction strokes to turn the alternator for more significant power generation.

Mechanical speed breakers can serve as both speed calming device and electricity generator, as evident in this work. Unwarranted speed has been identified as the major cause of road crashes (Agbonkhese et al., 2013; FRSC, 2014). Mechanical speed breaker electricity generator has the potential of regulating speed in over 132,000 km of local (truck “C”) roads across Nigeria, hence enhancing safety on the road. These roads have high vehicular density and limited mobility. The local roads connect communities and villages where the majority of the population live and conduct their business. A reasonable number of inhabitants engage in agricultural practice and small scale businesses. They use these roads for the movement of their goods. Improving safety on the road increase economic growth and reduce hazards hitherto faced by the road users.

Nigerian vehicular density is over 39 vehicles per kilometer (Federal Ministry of Works, 2013); therefore, the implementation of a mechanical speed breaker electricity generator will the boost electricity supply in the country, which is currently battling low-generation and epileptic electricity supplies. Access to the national grid supply is not always available in most communities in the nation and other remote power options are less viable. Currently, only about 36% of rural dwellers have access to the national grid. Implementing the mechanical speed breaker electricity generator in series with the distance of 120meters between one-speed breakers to another center to center will ensure sustainable electricity supply for street lighting and road signals. Also, electricity can be used to charge cartridge batteries for electric cars. The generation of power using a speed breaker is one of the promising future sources of energy as the human population and power requirement keep increasing. Likewise, the number of vehicles plying the roads keep increasing, resulting in higher vehicular density. Electricity can be generated throughout the year using mechanical speed breaker. Coupling this technology with a solar system, which is still undeveloped, will go a long way in solving the challenges of electricity supply in Nigeria. Cazzaniga et al. (2019) proposed the coupling of rapidly increasing renewable energy sources as a means of boosting electricity generation. Presently the significant sources of electricity in Nigeria are hydro and thermal plants, with thermal plants accounting for about 81% of total installed capacity. Nigeria, as a developing economy, can hinge her economic growth upon efficient generation and utilization of sustainable energy sources like mechanical speed breaker electricity generator and solar system (Sankaran et al., 2019). Mechanical speed breaker electricity generator does require the acquisition of a large area and is ecofriendly.

## 4 Conclusion

Although speed breakers are sometimes not accepted by some road users, they are veritable measures to improve safety on our road. Also, their application in generating electricity is an added advantage. They could be implemented on local (truck ‘C’) roads where speed limits are regulated. Truck “C” roads usually have high access and limited mobility. The energy generated could be used to light up the roads and power road signs. Sufficient illumination, especially at night, enhances visibility for road users and aids in combating criminal activities as most hoodlums perpetrate their evil acts under cover of darkness. The mechanical speed breaker was tested to generate 204W of electrical power per cycle. This work successfully designed and produced a double actuating mechanical speed breaker generator. Effective design and adequate material selection criteria were employed in the course of production of the machine. It was designed to be depressed by one wheel of an average saloon car weighing 1,600kg, while a prototype that can handle 400kg was constructed. The deflection of the springs was within acceptable limits, although excessive loading of the machine resulted in vibration. It is recommended that the full scale of the machine be installed on local roads in Nigeria to promote safety on the road and produce electricity to complement supply from the national grid.

## Declarations

### Author contribution statement

Jude Ezechi Dara, Christian Munachiso Odazie, Paul Chukwulozie Okolie & Andrew Onyemazuwa Azaka: Conceived and designed the experiments; Performed the experiments; Analyzed and interpreted the data; Wrote the paper.

### Funding statement

This research was supported by the Tertiary Education Trust Fund (TETfund), through Institution Based Research (IBR) grant; years 2012-2014 Merged TETFUND Research Projects (RP) Intervention, 8th Batch, 2017.

### Competing interest statement

The authors declare no conflict of interest.

### Additional information

No additional information is available for this paper.
